# The NOD2 receptor is crucial for immune responses towards New World *Leishmania* species

**DOI:** 10.1038/s41598-017-15412-7

**Published:** 2017-11-09

**Authors:** Jéssica Cristina dos Santos, Michelle S. M. A. Damen, Marije Oosting, Dirk J. de Jong, Bas Heinhuis, Rodrigo Saar Gomes, Carla Santos Araújo, Mihai G. Netea, Fátima Ribeiro-Dias, Leo A. B. Joosten

**Affiliations:** 1 Department of Internal Medicine and Radboud Center of Infectious Diseases (RCI), Radboud University Medical Center, Nijmegen, The Netherlands; 2 Instituto de Patologia Tropical e Saúde Pública, Universidade Federal de Goiás, Goiânia, Goiás Brazil; 3 Department of Gastroenterology and Hepatology, Radboud University Nijmegen Medical Center, Nijmegen, The Netherlands; 4 Universidade Federal do Vale do São Francisco, Petrolina, Pernambuco Brazil; 5 Human Genomics Laboratory, Craiova University of Medicine and Pharmacy, Craiova, Romania

## Abstract

American Tegumentary Leishmaniasis is a chronic infection caused by *Leishmania* protozoan. It is not known whether genetic variances in NOD-like receptor (NLR) family members influence the immune response towards *Leishmania* parasites and modulate intracellular killing. Using functional genomics, we investigated whether genetic variants in *NOD1* or *NOD2* influence the production of cytokines by human PBMCs exposed to *Leishmania*. In addition, we examined whether recognition of *Leishmania* by NOD2 contributes to intracellular killing. Polymorphisms in the *NOD2* gene decreased monocyte- and lymphocyte-derived cytokine production after stimulation with *L. amazonensis* or *L. braziliensis* compared to individuals with a functional NOD2 receptor. The phagolysosome formation is important for *Leishmania*-induced cytokine production and upregulation of *NOD2* mRNA expression. NOD2 is crucial to control intracellular infection caused by *Leishmania* spp. NOD2 receptor is important for *Leishmania* recognition, the control of intracellular killing, and the induction of innate and adaptive immune responses.

## Introduction

American Tegumentary Leishmaniasis (ATL) is a vector-borne parasitic disease caused by *Leishmania* protozoan that is characterized by lesions of the skin and oral or nasopharyngeal mucosa. Among the species of *Leishmania* and *Viannia* subgenus, *L*. (*L*.) *amazonensis* and *L*. (*V*.) *braziliensis* cause ATL leading to different clinical forms, which are dependent on the parasite species and the immune system of the host^1^. Both *L. amazonensis* and *L. braziliensis* can cause localized cutaneous leishmaniasis (LCL). In the most severe cases of Leishmaniasis, *L. amazonensis* can cause diffuse cutaneous lesions (DCL) and *L. braziliensis* can cause mucocutaneous lesions (ML)^2^.

Innate immune cells such as macrophages, neutrophils, and natural killer cells recognize microorganisms through interaction between microbial ligands (MAMPs) with their pattern recognition receptors (PRRs). These PRRs include Toll-like receptors (TLRs) and NOD-like receptors (NLRs)^3,4^. The *Leishmania* membrane has lipophosphoglycans (LPG), glycoinositolphospholipids (GIPLs), and glycoprotein 63 (gp63) as the main molecules that are recognized by innate immune cells^5,6^. Recent studies have shown the involvement of TLRs in recognizing some of these molecules. LPG and GIPLs are recognized by TLR4 and TLR2, respectively. TLR9 also plays a role in recognizing the DNA of *Leishmania* spp. These mechanisms lead to induction of cytokines and microbicidal molecules after exposure to *Leismania* spp.^7–11^. In addition to, NLRs are important receptors for recognition of several microorganisms^12^. However, few studies have described their role in *Leishmania* recognition. Lima Junior *et al* ^13^ have shown that the NLRP3 inflammasome plays an important protective role during *L. amazonensis* infection in a mouse model. Conversely, it was showed that NLRP3 activation followed by IL-1β production mediates the detrimental CD8^+^ T cell-mediated cytotoxicity in tegumentary leishmaniasis, causing lesions/chronic inflammation in mouse model of infections caused by *L. braziliensis* or *L. major* ^14^. NLRP3 can also contribute to mouse susceptibility to *L. major* by increasing neutrophils recruitment, which plays a crucial role in the development of nonhealing lesions^15^. Recently, in patients with visceral leishmaniasis and in a murine infection model with *L. infantum*, the NOD2-RIPK2 pathway has been found to be involved in the development of the Th1-type response^16^, the most important response against *Leishmania* spp. Because the innate immune response is important in driving the acquired immune response, the efficient recognition of the parasite by PRRs improves the host resistance^16^.

*Leishmania* recognition by monocytes or macrophages through TLRs leads to the production of proinflammatory cytokines such as tumor necrosis factor (TNFα), interleukin (IL)-6, and interferon gamma (IFNγ), which all contribute for the exacerbated inflammation in leishmaniasis lesions^11,17–19^. Following NLRP3 inflammasome activation, IL-1β can be produced and contributes to the control of murine *Leishmania* infection^13^. Besides inflammation, these proinflammatory cytokines are important for controlling the *Leishmania* infection by inducing microbicidal molecules, such as reactive oxygen and nitrogen intermediates (ROI, RNI), which are crucial for the parasite killing^10,19–22^.

Since it is known that the immunogenetic background of the patients is one of the most important factors to determine the clinical outcome of leishmaniasis^23^, we investigated the role of genetic variants in genes of the NLR family for the *Leishmania*-induced immune response.

## Results

### Individuals heterozygous for *NOD2* mutation produce less cytokines after *Leishmania* spp. exposure compared to individuals with wild-type *NOD2*

**Figure 1:**
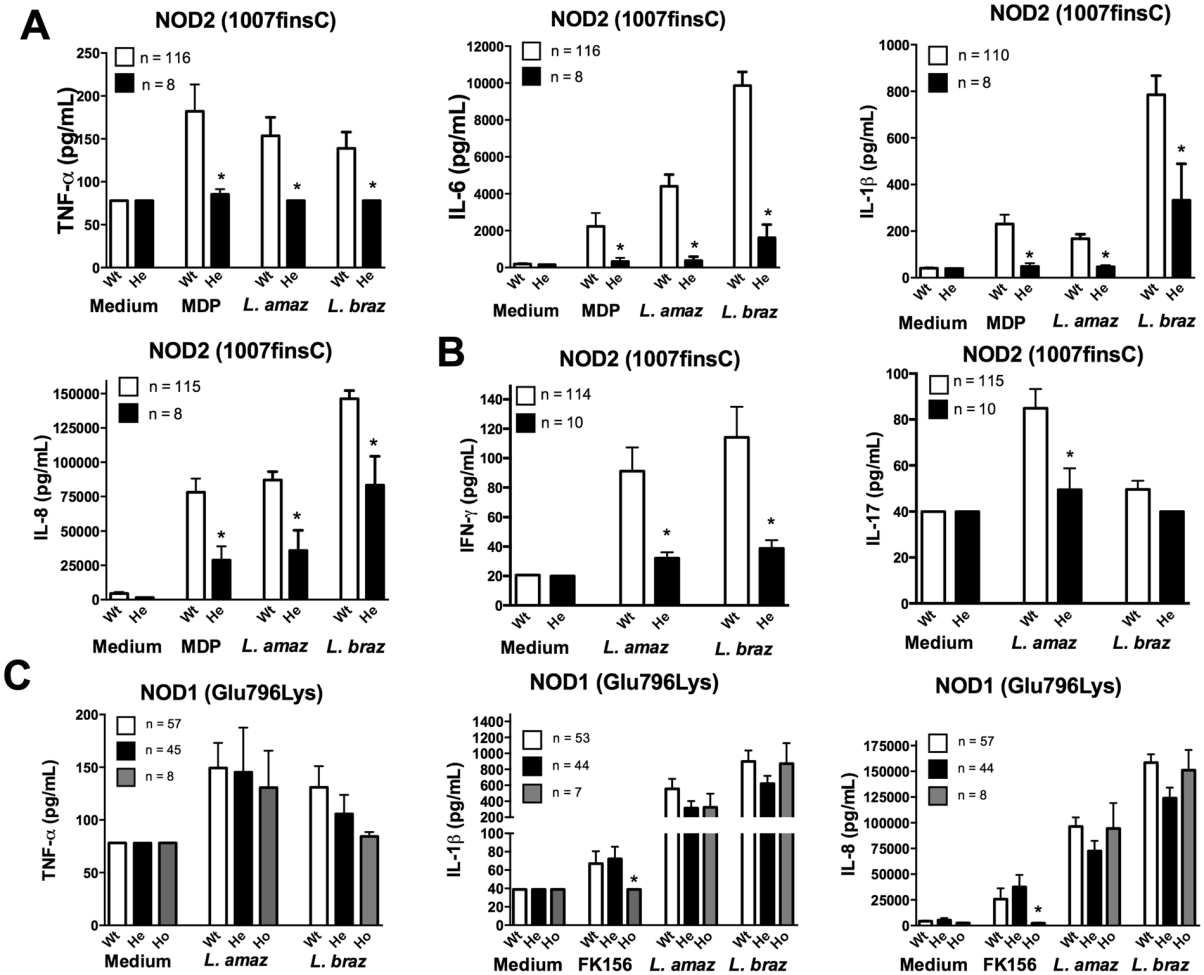
*NOD2* but not *NOD1* plays an important role in monocyte-and lymphocyte-derived cytokines induced by after *Leishmania* pecies stimulation. Peripheral blood mononuclear cells (PBMCs, 5 × 10^5^ cells/100 μL) from healthy individuals genotyped for *NOD1* (Glu796Lys) and *NOD2* (1007finsC) were stimulated with different stimuli including lysates of *Leishmania* spp. (50 μg/mL, *L*. (*L*.) *amazonensis*: *L. amaz*; *L*. (*V*.) *braziliensis*: *L. braz*), FK156 (10 μg/mL), MDP (10 μg/mL); Medium: non-stimulated cells. TNFα, IL-6, IL-1β and IL-8 concentrations were measured in supernatants by ELISA after 24 h of incubation. IFNγ and IL-17 were determined after 7 days of incubation: the *NOD2* (**A** and **B**) and *NOD1* (**C**) genotype. Bars represent individuals carrying no SNP (wild type, Wt, white bars), heterozygous SNP carries (He, black bars), or homozygous variation (Ho, grey bars). Data represent the mean ± SEM. *p < 0.05; Mann-Whitney U-test (**A** and **B**; Wt vs He); (**C**; Wt vs Ho).

We explored whether SNPs in NLR family members NOD1 and NOD2 influence the production of cytokines after stimulation with *Leishmania* parasites. Through these analyses, we found that the NOD2 receptor plays an important role in the immune response against *Leishmania* spp. (Fig. 1). The results demonstrated that individuals heterozygous for *NOD2* Leu1007insC polymorphism displayed significantly lower production of TNFα, IL-1β, IL-6, IL-8 and IFNγ for either *L*. *amazonensis* or *L. braziliensis* stimulation (Fig. 1A,B). Interestingly, NOD2 was only important for IL-17 induced by *L*. *amazonensis*, while *L. braziliensis* practically did not induce IL-17 production (Fig. 1B). Confirming the relevance of this *NOD2* mutation for cytokine induction, the NOD2 agonist MDP induced lower cytokine production when added to PBMCs from individuals with the Leu1007insC variance, compared to the control subjects (Fig. 1A). Evaluation of other common polymorphisms in the *NOD2* gene revealed no differences in the *Leishmania*-induced cytokine production (Figs S1 and 2). In addition to *NOD2*, we also investigated whether the *NOD1* receptor could play a role in the cytokine induction after *Leishmania* exposure. In contrast to NOD2, no differences in monocyte- or lymphocyte-derived cytokine production were noted when PBMCs homozygous for *NOD1* Glu796Lys were stimulated with *L. amazonensis* or *L. braziliensis* (Fig. 1C). Using the NOD1 agonist FK156, we demonstrated that Glu796Lys variance results in loss of function of NOD1 (Fig. 1C).

### Phagolysosome formation is important for *Leishmania*-induced cytokine production and upregulation of *NOD2* mRNA expression

**Figure 2:**
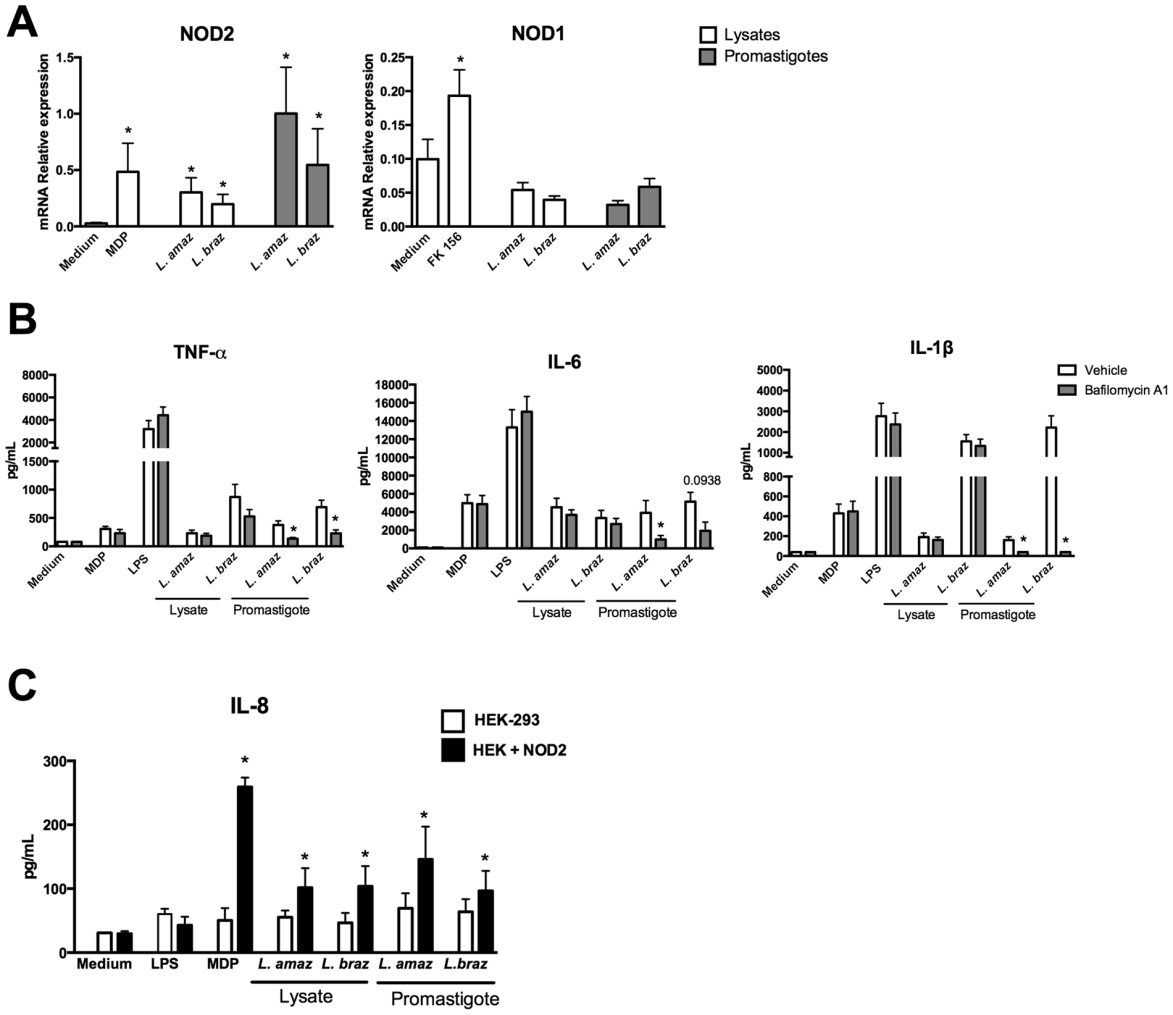
*Leishmania* species induce *NOD2* mRNA expression and phagolysosome formation is important for *Leishmania*-induced cytokine production. (**A** and **B**) Peripheral blood mononuclear cells (PBMCs, 5 × 10^5^ cells/100 μL) from healthy individuals were stimulated with either lysates (50 μg/mL) or promastigotes (1 × 10^5^ parasites) of *Leishmania* species (*L*. (*L*.) *amazonensis*: *L. amaz*; *L*. (*V*.) *braziliensis*: *L. braz)*; Medium (non-stimulated cells), FK156 (10 μg/mL), MDP (10 μg/mL) and LPS (10 ng/mL) were used as controls. (**B**) In some experiments cells were incubated in the absence (white) or presence (gray) of bafilomycin A1 (250 nM). Medium plus Vehicle (DMSO). (**A**) *NOD1* and *NOD2* mRNA expression were determined by quantitative real-time PCR after 24 h of incubation. (**B**) TNFα, IL-6 and IL-1β concentrations were determined in supernatants by ELISA after 24 h of incubation. Data represent the mean ± SEM, *p < 0.05; Wilcoxon test (**A**) (Medium vs stimuli) (**B**) (Vehicle vs Bafilomycin) (n = 6, in 2 independent experiments done in duplicates). (**C**) Embryonic kidney (HEK)-293 cells (1 × 10^6^ cells/100 µL) transfected or not with *NOD2* were stimulated for 24 h with lysates of *Leishmania* species (50 μg/mL) or promastigote forms (2 × 10^6^ parasites) in the stationary growth phase of *L. amaz*; *L. braz*. Medium, LPS (10 ng/mL), and MDP (10 μg/mL) were included as controls. Protein levels of IL-8 were determined by ELISA in supernatants. Data represent the mean ± SEM of three independent experiments, *p < 0.05; Paired t-test (HEK-293 vs HEK + NOD2).

In order to investigate whether *Leishmania* could upregulate the mRNA expression of *NOD2*, human PBMCs were incubated for 24 h with either parasite lysates or live promastigote forms of both *L. amazonensis* or *L. braziliensis*. The use of both forms of *Leishmania* stimuli was determined by the need to examine whether NOD2 is activated by *Leishmania* fragments which means that the parasites needs to be degraded intracellularly. Figure 2A showed that lysates as well as intact parasites of both *Leishmania* spp. were able to increase the *NOD2* mRNA expression but not *NOD1* mRNA. As positive controls FK-156 and MDP both could upregulation *NOD1* or *NOD2* mRNA expression respectively (Fig. 2A).

To examine whether degradation of the parasite by the phagolysosome is essential for *Leishmania*-induced cytokine production we inhibited the formation of phagolysosomes. PBMCs were incubated in the absence or presence of bafilomycin A1, a chemical compound that prevents maturation of parasitophorus vacuoles by inhibiting fusion between phagosomes and lysosomes^24^. Figure 2B shows a remarkable decrease in TNFα and IL-1β production after exposure to promastigote forms of either *L.amazonensis* and *L. braziliensis* in the presence of bafilomycin A1 (Fig. 2B). For IL-6, a decrease was observed after exposure to *L. amazonensis* and despite a tendency to reduction after exposure to *L. braziliensis* the difference did not achieve statistical significance (p = 0.0938). No differences in TNFα, IL-1β or IL-6 concentrations were observed when lysates of both *Leishmania* spp. were used to activate PBMCs in absence or presence of bafilomycin A1 (Fig. 2B).

To confirm the pivotal role of NOD2 in the recognition of *Leishmania*, HEK-293 cells overexpressing NOD2 were exposed to either lysates or live promastigote forms of either *L. amazonensis* or *L. braziliensis*. We observed a strong increase in IL-8 production after stimulation with either lysates or promastigote forms of *Leishmania* pecies when NOD2 was present in the HEK-293 cells (Fig. 2C). A similar effect in IL-8 production was observed when NOD2 agonist MDP was added as a positive control. A potent TLR4 agonist (LPS) was used as negative control, showing no elevated IL-8 production. To validate the HEK-293-NOD2 reporter cells, we determined *NOD2* mRNA expression before and after MDP exposure by quantitative real-time PCR (Fig. S3).

### PBMCs isolated from patients bearing *NOD2* (3020insC) mutation confirmed the crucial role of NOD2 for *Leishmania*-induced cytokine production

**Figure 3:**
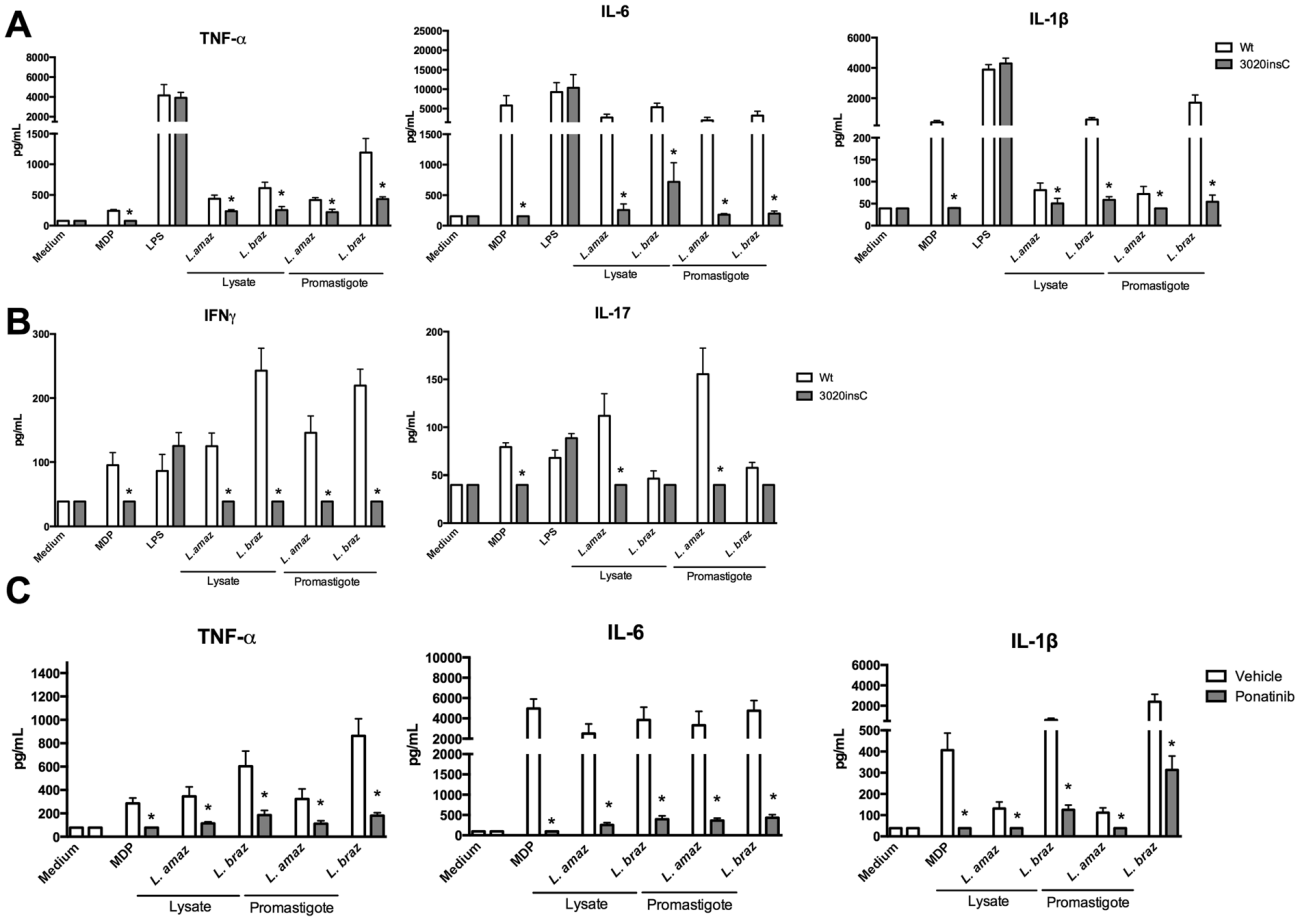
*NOD2* plays an important role in the cytokines after *Leishmania* stimulation. (**A**,**B**) Peripheral blood mononuclear cells (PBMCs, 5 × 10^5^ cells/100 μL) from healthy individuals (n = 8) carrying no mutation in *NOD2* (3020insC) (Wt; white bars) and from individuals carrying a mutation in *NOD2* receptor (n = 4; grey bars) were stimulated with either lysates (50 μg/mL) or promastigotes (1 × 10^5^ parasites) of *Leishmania* species (*L*. (*L*.) *amazonensis*: *L. amaz*; *L*. (*V*.) *braziliensis*: *L. braz)*; Medium (non-stimulated cells), MDP (10 μg/mL) and LPS (10 ng/mL) were used as controls. (**A**) TNFα, IL-6 and IL-1β concentrations were determined in supernatants by ELISA after 24 h incubation. (**B**) IFNγ and IL-17 were determined after 7 days of incubation in supernatants by ELISA; Data represent the mean ± SEM, *p < 0.05; Mann-Whitney U-test (Wt vs 3020insC). (**C**) Peripheral blood mononuclear cells (PBMCs, 5 × 10^5^ cells/100 μL) from healthy individuals were stimulated with either lysates (50 μg/mL) or promastigotes (1 × 10^5^ parasites) of *Leishmania* species (*L*. (*L*.) *amazonensis*: *L. amaz*; *L*. (*V*.) *braziliensis*: *L. braz)* in the presence (grey bars) or absence (white bars) of the Ponatinib (100 nM); Medium (non-stimulated cells) plus Vehicle (DMSO) and MDP (10 μg/mL) were used as controls. TNFα, IL-6 and IL-1β concentrations were determined by ELISA, after 24 h incubation. Data represent the mean ± SEM, *p < 0.05; (DMSO vs Ponatinib; n = 6, in 2 independent experiments done in duplicates, by Wilcoxon test).

Additional experiments were performed to confirm the role of NOD2 in *Leishmania* recognition. To this end, PBMCs carrying the *NOD2* frameshift (3020insC) mutation were stimulated with live parasites or lysates of parasites for 24 h. The production of cytokines was compared with PBMCs isolated from healthy individuals (wt). As expected, cytokine concentrations were complete absent in individuals homozygous for the 3020insC frameshift mutation when PBMCs where stimulated with MDP (Fig. 3A). LPS-induced cytokine production was similar between PBMCs isolated from subjects carrying the 3020insC frameshift mutation and healthy controls (Fig. 3A). Production of TNFα, IL-1β, IL-6, IL-8, IFNγ was significantly decreased in PBMCs bearing the *NOD2* 3020insC frameshift mutation compared to PBMCs of individuals without this mutation after exposure to lysates or promastigote forms of both *L. amazonensis* and *L. braziliensis* (Fig. 3A and B). Of high interest, a reduction of IL-17 production was only observed after exposure to *L. amazonensis* (Fig. 3B).

To investigate the downstream pathway of NOD2 signalling, we stimulated human PBMCs in the absence or presence of Ponatinib, a drug that potently inhibits the phosphorylation of RIPK2^25^. RIPK2 is the important kinase for the NOD2 signalling cascade^26^. A significant reduction in TNFα, IL-1β and IL-6 production was observed when PBMCs were incubated with lysates or promastigote forms of both *L. amazonensis* and *L. braziliensis* in the presence of Ponatinib (Fig. 3C). A dose-response experiment with Ponatinib was performed and no cytotoxicity of Ponatinib to human cells or parasites was found (Fig. S4).

### NOD2 is important to control intracellular infection caused by *Leishmania* spp

**Figure 4:**
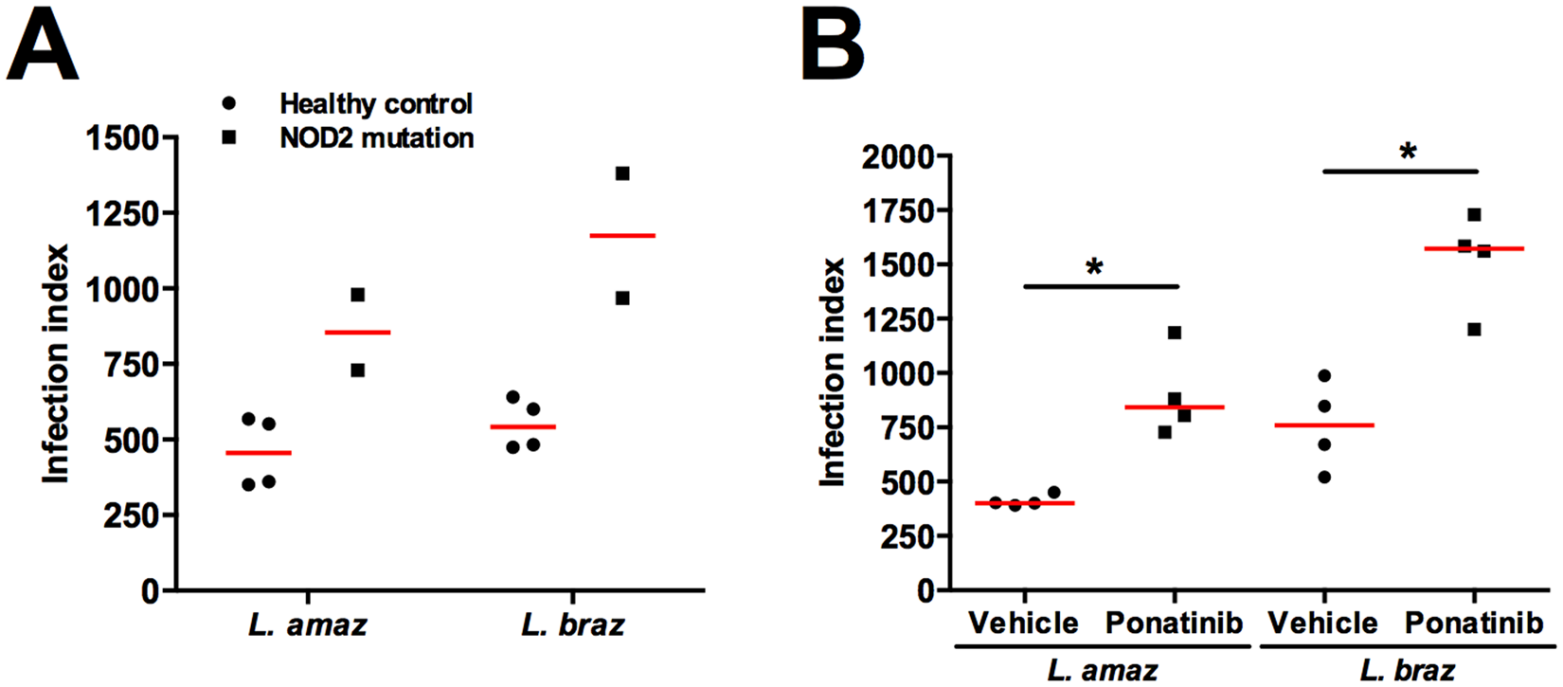
NOD2 plays a role in control of Leishmania spp. infection. Monocyte-derived macrophages were obtained from PBMCs from wild type (wt) individuals (n = 4) carrying no mutations in *NOD2* receptor (3020insC) (black circles) and individuals carrying mutations in *NOD2* (n = 2, black squares) (**A**), after 5 days of differentiation. On day 5, macrophages (5 × 10^5^ cells) were infected with promastigote forms of either *L*. (*V*.) *braziliensis* and *L*. (*L*.) *amazonensis* (2.5 × 10^5^ parasites) during 4 h. (**B**) Macrophages from wt individuals (n = 4) were incubated in the absence (black balls - Vehicle DMSO) or presence (black squares) of Ponatinib (100 nM) 1 h before. Cells were washed out to remove the non-internalized parasites after 4 h, Ponatinib was added again and cells were incubated for 48 h. Coverslips were collected to determine macrophage infection index. Data represent individual values and horizontal lines represent medians. *p < 0.05; Wilcoxon test (DMSO vs Ponatinib).

We investigated whether NOD2 plays a role in controlling *Leishmania* spp. infection in human primary macrophages. To this end, monocyte-derived macrophages from individuals homozygous for the *NOD2* 3020insC polymorphism were compared with macrophages from healthy individuals. Both types of macrophages were infected with either promastigote forms of *L. amazonensis* or *L. braziliensis*. Figure 4A shows an increase of the macrophage infection index in cells isolated from individuals carrying the *NOD2* loss-of-function mutation compared with healthy controls (wt). Similar results were found in primary human macrophages pre-treated with Ponatinib, which showed an increase in the infection index compared to vehicle-treated macrophages (Fig. 4B). The results demonstrated that the NOD2 receptor plays an important role in the control of *Leishmania* infection in human macrophages.

## Discussion

In the present study, we investigated the role of the NLR family members NOD1 and NOD2 in the induction of cytokines in human PBMCs after exposure to New World *Leishmania* spp. Results showed that whereas NOD2 plays an important role in parasite-induced cytokines, NOD1 is not relevant. Moreover, NOD2 is important for microbicidal activity of human macrophages contributing to the control of *Leishmania* spp. infection. This is the first report describing the NOD2 involvement in activation of human cells by New World *Leishmania* spp. Recently, a study in mice with *L. infantum* demonstrated that the activation of NOD2-RIP2 pathway drives the development of a Th1 instead of Th17 immune response^16^. These results demonstrated an important role of NOD2 in controlling *Leishmania* infection.

SNPs in the *NOD2* receptor are related to autoinflammatory diseases such as Crohn’s disease and Blau syndrome^27–29^. In general, as a consequence, individuals carrying these mutations have a reduced NF-κB activation after recognition of specific pathogen-associated NOD2 ligands, which interfere in cytokine production^30^. Mutations in *NOD1* lead to a reduced capacity to detect their ligands^31^. To evaluate the role of genetic variance in *NOD1* and *NOD2* in the immune response to *Leishmania* antigens, we applied a functional genomics approach as reported recently^32–34^. Using whole exome sequencing data of 100 subjects, we selected SNPs that are frequently present in *NOD1* or *NOD2* with a predicted loss or gain of function^35^. Here, we showed that one particular genetic variance in *NOD2* (Leu1007finsC) downregulates the production of monocyte-derived cytokines such as TNFα, IL-1β, IL-6, IL-8 as well as IFNγ after exposure to lysates of either *L. amazonensis* or *L. braziliensis*. Remarkably, IL-17 was induced after exposure to lysates of *L. amazonensis*, but not *L. braziliensis* in a NOD2-dependent manner. The results indicate that in addition to its role in activation of the innate immunity, NOD2 shapes the adaptive immune response against *Leishmania* spp. Our results are partially in agreement with the results demonstrated by Nascimento *et al*.^16^, in which, the authors showed that in a murine model of *L. infantum* infection, the development of Th1 responses and the production of IFNγ was dependent on NOD2. However, the authors showed that the NOD2 pathway was not relevant for Th17 development. In contrast, we demonstrated that in human PBMCs, IL-17 production is NOD2-dependent only after exposure to *L. amazonensis*. These results could be ascribed to the differences among *Leishmania* spp. as well as to differences in human and mouse immune responses^36^. Here, besides *Leishmania* antigens (parasite lysates) or live promastigotes increased cytokines in a NOD2-dependent manner they upregulated *NOD2*, but not *NOD1* mRNA expression in PBMCs. These results strengthened the involvement of NOD2 but not NOD1 in *Leishmania* spp.-induced immune responses.

To confirm that NOD2 is relevant for *Leishmania*-induced cytokine production, we overexpressed NOD2 in HEK-293 cells and showed that these NOD2-transfected cells produced significantly higher IL-8 concentrations after exposure to parasite lysates or promastigote forms of either *L. amazonensis* or *L. braziliensis*, compared to control HEK-293 cells. Although, NOD2 is known as a receptor that recognizes structures present in the bacteria cell wall^37,38^, the results presented here suggest that the NOD2 receptor also recognizes protozoan structures.

It is well described that PBMCs from patients with Crohn’s disease carrying the *NOD2* (*NOD2* 3020insC) mutation display a reduction in cytokine production after exposure to several microbial ligands or pathogens^39,40^. In our study, we also used PBMCs from subjects carrying *NOD2* 3020insC mutation and demonstrated a significant decrease in the production of proinflammatory cytokines after *Leishmania* spp. exposure. These data are in line with the results of the *NOD2* genetic variance Leu1007finsC and the HEK-NOD2 cells, underlining the pivotal role of NOD2 in the recognition of *Leishmania* spp.

An additional line of evidence that the NOD2 pathway is important for *Leishmania*-induced immune responses, are our results with the RIPK2 inhibitor (Ponatinib). The inhibition of RIPK2 led to almost complete abolishment of *Leishmania* cytokine production. These data demonstrate previously unknown role for human NOD2 in the recognition of *L. amazonensis* or *L. braziliensis* and make it tempting to speculate that individuals carrying *NOD2* polymorphisms might be more susceptible to ATL.

The ligand of *Leishmania* parasites that binds to NOD2 remains unknown but we showed that degradation of *Leishmania* is a prerequisite for cytokine production. Bafilomycin A1, a specific inhibitor of the phagosome acidification and blocker of phagosome maturation impaired the proinflammatory cytokine production only after exposure to live promastigote forms of *L. amazonensis* or *L. braziliensis*, whereas no effect was observed after exposure to lysates of both these species. Enzymes present in the phagolysosomes, such as lysozyme, may degrade *Leishmania* to release NOD2 ligands in a similar process that happens with *Listeria monocytogenes* ^41^. Future studies are warranted to investigate which microbial components of *Leishmania* are recognized by NOD2.

It is known that the production of proinflammatory cytokines can play a double role in infection caused by *Leishmania* spp., contributing to the control of the infection but also favoring inflammation and tissue destruction^42,43^. Here we demonstrated that loss of NOD2 signalling impairs intracellular *Leishmania* killing. The exact mechanisms involved in NOD2-mediated *Leishmania* killing need to be further explored, although it is known that proinflammatory cytokines promote the induction of microbicidal molecules after infection with *Leishmania* spp.^10,19–22,44^. Moreover, it is known that NOD2 plays an important role in induction of microbicidal mechanisms including autophagy, antimicrobial peptides and ROS production, which are essential to control infections caused by intracellular pathogens^45–47^.

**Figure 5:**
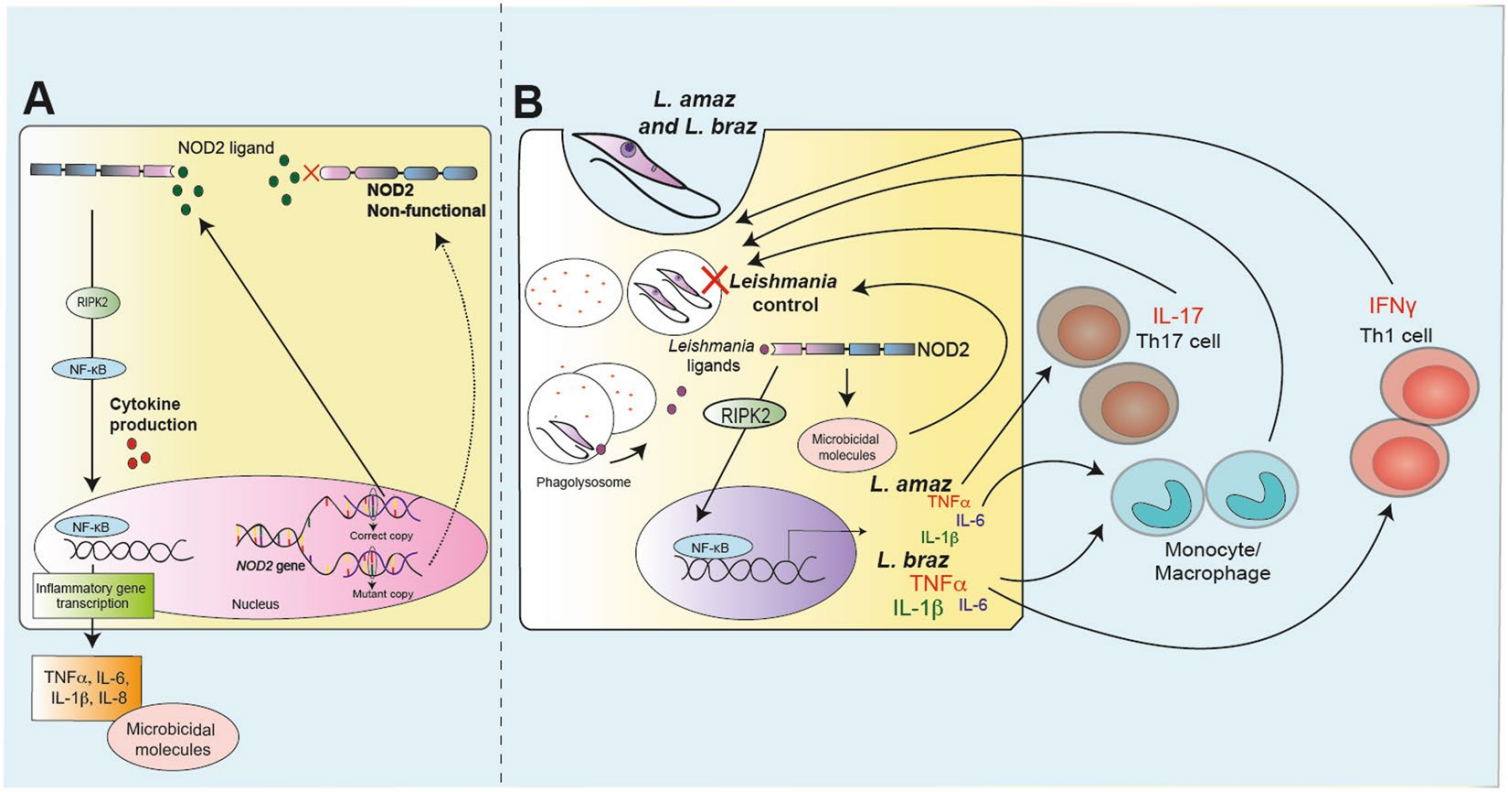
Schematic overview of the role of NOD2 receptor in *Leishmania* spp. recognition and control. (**A**) General overview of NOD2 pathway: The NOD2 receptor is engaged by its respective ligand, driving the activation of RIPK2, which is followed by NF-κB activation and translocation to the nucleus leading to inflammatory gene transcription. In addition, microbicidal molecules can be produced. NOD2 genetic variances can lead to a non-functional NOD2 receptor, as a consequence, the cytokine and microbicidal molecule production mediated by NOD2 can be affected. (**B**) Phagolysosome-mediated degradation of *Leishmania* spp. promastigote forms leads to parasite antigen release in the cytoplasm of infected cells. This may drive the NOD2 activation and subsequently induction of NF-κB gene transcription and microbicidal molecule production. *L*. (*L*.) *amazonensis* and *L*.(*V*.) *braziliensis*-NOD2-mediated proinflammatory cytokines produced by innate and adaptive cells might contribute for controlling of the infection.

To conclude, our study showed for the first time that the human NOD2 pathway is important for *Leishmania* recognition, the induction of innate and adaptive immune responses, and the control of intracellular killing of the parasite (Fig. 5). Our findings provide the first evidence that genetic variances in NOD2 are associated with differences in the human immune response towards *Leishmania* spp. The relevance of the NOD2 pathway in the susceptibility to or severity of human ATL needs to be investigated in the near future.

## Methods

### Ethics Statement

The study was approved by Ethics Committee of Radboud University Nijmegen, the Netherlands (no. 42561.091.12). Experiments were conducted according to the principles expressed in the Declaration of Helsinki. All individuals gave written informed consent to donate blood.

### 200FG cohort

Healthy individuals with a Dutch European genetic background that were recruited as part of the 200FG study^34^ participated in the study. The volunteers were between 23–73 years old, and consisted of 77% men and 23% women.

### Isolation of genomic DNA and single-nucleotide polymorphism (SNP) analysis

DNA was isolated from whole blood by using the isolation Gentra Pure Gene Blood kit (Qiagen), according to the manufacturer’s protocol. SNPs in the analyzed receptor genes were selected from the National Center for Biotechnology Information SNP database (http://www.ncbi.nlm.nih.gov/snp/) upon previously described associations with human diseases and a minor allele frequency of at least 5% among different populations. In total, 7 SNPs in *NOD1* and *NOD2* receptors were selected and genotyped (Supplementary Table 1). Gene fragments were amplified by commercially available TaqMan® SNP Genotyping Assays according to the manufacturer’s protocol on the AB StepOnePlus polymerase chain reaction system (Applied Biosystems). Quality control was performed by the incorporation of positive and negative controls and duplication of random samples across different plates.

### *Leishmania* cultures and lysates

*L. amazonensis* (IFLA/BR/67/PH8) reference strain and MHOM/BR/2003/IMG *L. braziliensis*, a clinical isolate obtained from a LCL patient (Leishbank IPTSP/UFG)^48^, were used. Promastigote forms were cultured in Grace’s Insect Medium, (Gibco - Life Technologies) and prepared for experiments as described by dos Santos *et al*.^19^. To obtain lysates of *Leishmania*, promastigotes (1 × 10^9^ cells/mL) were lysed by 5 freeze-thaw cycles in liquid nitrogen and water bath at 37 °C followed by protein quantification using the Pierce BCA protein assay (Thermo Scientific). For cell stimulations, parasites were suspended in RPMI 1640 medium (Sigma) and added to the cultures as described below.

### Human embryonic kidney cell line (HEK) stimulation

Transfection of HEK-293 cells with human *NOD2* was performed as previously described by Laayouni *et al*.^49^ and culture conditions are described as supplementary material. Non-transfected or NOD2-transfected HEK-293 cells (1 × 10^6^ cells) were added to 96-well flat-bottom plates (Greiner) in the presence of either *E. coli* lipopolysaccharide (LPS, O111:B4, 10 ng/mL; Sigma), further purified based on^50^, muramyl dipeptide (MDP, 10 μg/mL; Sigma), lysates or promastigotes (2 × 10^6^ parasites) of *L. braziliensis* or *L. amazonensis* in a final volume of 200 μL. After 24 h of incubation, at 37 °C and 5% CO_2_, supernatants were collected and stored at −20 °C until analysis of IL-8 production.

### Isolation of human peripheral blood mononuclear cells (PBMC) and treatments

Venous blood was obtained from eight healthy individuals bearing the wild-type allele of *NOD2* (wild type, wt) and from four homozygous individuals for the *NOD2* 3020insC mutation presenting Crohn’s disease. Isolation of PBMCs was performed as described previously^39^, by density gradient centrifugation of blood diluted 1:1 in pyrogen-free saline overlayed on Ficoll-Paque (Pharmacia Biotech). PBMCs were suspended in RPMI 1640 medium with 50 mg/mL gentamicin, 2 mmol/L L-glutamine, and 1 mmol/L pyruvate (Invitrogen). The number was adjusted to 5 × 10^6^ cells/mL. Cells (5 × 10^5^ cells/mL) were added to round-bottom 96-well plates (Greiner) and incubated with either culture medium (negative control), MDP (10 μg/ml), FK-156 (10 μg/mL, Sigma), ultra-pure *E. coli* LPS as described by Battisti & Minnick^50^ (O111:B4 serotype, 10 ng/mL; Sigma) or 50 μg/mL of both *Leishmania* pp. lysates or promastigotes (1 × 10^5^ parasites) of *L. braziliensis* and *L. amazonensis* in the presence or absence of 10% of human pool serum. In some experiments, PBMCs were pre-incubated for 1 h with a RICK inhibitor (Ponatinib, 100 nM/mL; Selleckchem) and a phagolysosome inhibitor (Bafilomycin A1, 250 nM; Invivogen) before cells were stimulated with lysates or promastigotes. After 24 h or 7 days, the supernatants were collected and stored at −20 °C until further analysis. The cell monolayers were collected by adding 200 µL of TRIzol and stored at −80 °C until being used for mRNA extraction.

### Measurement of cytokines

Human TNFα, IL-1β, IL-6, IL-8, IL-17A and IFNγ were determined in culture supernatants using commercial Enzyme-Linked Immunosorbent Assay (ELISA) kits (Sanquin, Amsterdam, and R&D Systems, Minneapolis), according to the manufacturer’s protocols.

### Evaluation of mRNA expression by quantitative real-time PCR (qPCR)

RNA isolation was carried out based on the method reported by Chomzynski & Sacchi^51^ and the qPCR method is shortly described in supplementary material. Primer sequences (Supplementary Table 2) for *NOD1* and *NOD2* receptors were obtained from the Harvard Primerbank database. Primers were purchased from Biolegio. Relative expression of mRNA levels was calculated and normalized for the housekeeping gene GAPDH.

### Evaluation of Macrophage infection

Monocyte-derived macrophages from healthy volunteers and from homozygous individuals for the *NOD2* 3020insC mutation were obtained from PBMCs (5 × 10^5^ cells/well) during 5 days at 37 °C, 5% CO_2_. PBMCs were counted and plated into 24-well plates (Costar) over coverslips in the presence of RPMI-1640 medium supplemented as described above and added with 10% of human pool serum. Medium was refreshed every 48 h. On day 5, macrophages were infected with promastigote forms of either *L. braziliensis* or *L*. *amazonensis* (2.5 × 10^5^ parasites) during 4 h. Cells were washed to remove non-internalized parasites and incubated for 48 h. Additionally, is some experiments monocyte-derived macrophages were pre-treated or not with Ponatinib (100 nM/mL; Selleckchem) 1 h before infection and the drug was replaced after washings. Coverslips were collected to determine the macrophage infection index after cells were fixed and stained with Giemsa (Merck Millipore), according to^52^ and briefly described in supplementary material.

### Statistical Analysis

Data are expressed as median, interquartile, minimal and maximal values, unless otherwise indicated. Differences between experimental groups were tested by Mann-Whitney *U* test or unpaired t test according to the data, or by Wilcoxon paired test, using Prism software (version 6.0; GraphPad; San Diego, CA, USA). Differences with *p* < 0. 05 were considered significant.

## Electronic supplementary material

Supplementary information

## References

[1] Gontijo B, de Carvalho MdeLR (2003). [American cutaneous leishmaniasis]. Rev. Soc. Bras. Med. Trop..

[2] Reithinger R (2007). Cutaneous leishmaniasis. Lancet. Infect. Dis..

[3] Kumar H, Kawai T, Akira S (2009). Pathogen recognition in the innate immune response. Biochem. J..

[4] Moreira LO, Zamboni DS (2012). NOD1 and NOD2 Signaling in Infection and Inflammation. Front. Immunol..

[5] Turco SJ, Sacks DL (2003). Control of Leishmania-sand fly interactions by polymorphisms in lipophosphoglycan structure. Methods Enzymol..

[6] McConville, M. J. & Ferguson, M. A. The structure, biosynthesis and function of glycosylated phosphatidylinositols in the parasitic protozoa and higher eukaryotes. *Biochem. J*. 305–24 (1993). 10.1042/bj2940305 PMC1134455 8373346

[7] Becker I (2003). Leishmania lipophosphoglycan (LPG) activates NK cells through toll-like receptor-2. Mol. Biochem. Parasitol..

[8] Liese J, Schleicher U, Bogdan C (2007). TLR9 signaling is essential for the innate NK cell response in murine cutaneous leishmaniasis. Eur. J. Immunol..

[9] Assis RR, Ibraim IC, Noronha FS, Turco SJ, Soares RP (2012). Glycoinositolphospholipids from Leishmania braziliensis and L. infantum: modulation of innate immune system and variations in carbohydrate structure. PLoS Negl. Trop. Dis..

[10] Ibraim IC (2013). Two biochemically distinct lipophosphoglycans from Leishmania braziliensis and Leishmania infantum trigger different innate immune responses in murine macrophages. Parasit. Vectors.

[11] Nogueira PM (2016). Lipophosphoglycans from Leishmania amazonensis Strains Display Immunomodulatory Properties via TLR4 and Do Not Affect Sand FlyInfection. PLoS Negl. Trop. Dis..

[12] Davis BK, Wen H, Ting JP-Y (2011). The Inflammasome NLRs in Immunity, Inflammation, and Associated Diseases. Annu. Rev. Immunol..

[13] Lima-Junior DS (2013). Inflammasome-derived IL-1β production induces nitric oxide–mediated resistance to Leishmania. Nat. Med..

[14] Novais FO (2017). CD8+ T cell cytotoxicity mediates pathology in the skin by inflammasome activation and IL-1β production. PLoS Pathog..

[15] Charmoy M (2016). The Nlrp3 inflammasome, IL-1β, and neutrophil recruitment are required for susceptibility to a nonhealing strain of *Leishmania major* in C57BL/6 mice. Eur. J. Immunol..

[16] Nascimento MSL (2016). NOD2-RIP2–Mediated Signaling Helps Shape Adaptive Immunity in Visceral Leishmaniasis. J. Infect. Dis..

[17] Faria MS (2014). Role of protein kinase R in the killing of Leishmania major by macrophages in response to neutrophil elastase and TLR4 via TNF and IFN. FASEB J..

[18] Galdino H (2016). Leishmania (Viannia) braziliensis amastigotes induces the expression of TNFα and IL-10 by human peripheral blood mononuclear cells *in vitro* in a TLR4-dependent manner. Cytokine.

[19] dos Santos JC (2017). Cytokines and microbicidal molecules regulated by IL-32 in THP-1-derived human macrophages infected with New World Leishmania species. PLoS Negl. Trop. Dis..

[20] Díaz NL, Arveláez FA, Zerpa O, Tapia FJ (2006). Inducible nitric oxide synthase and cytokine pattern in lesions of patients with American cutaneous leishmaniasis. Clin. Exp. Dermatol..

[21] Carvalho LP, Passos S, Schriefer A, Carvalho EM (2012). Protective and pathologic immune responses in human tegumentary leishmaniasis. Front. Immunol..

[22] Carneiro PP (2016). The Role of Nitric Oxide and Reactive Oxygen Species in the Killing of Leishmania braziliensis by Monocytes from Patients with Cutaneous Leishmaniasis. PLoS One.

[23] Sophie M (2017). SLC11A1 polymorphisms and host susceptibility to cutaneous leishmaniasis in Pakistan. Parasit. Vectors.

[24] Yamamoto A (1998). Bafilomycin A1 prevents maturation of autophagic vacuoles by inhibiting fusion between autophagosomes and lysosomes in rat hepatoma cell line, H-4-II-E cells. Cell Struct. Funct..

[25] Canning P (2015). Inflammatory Signaling by NOD-RIPK2 Is Inhibited by Clinically Relevant Type II Kinase Inhibitors. Chem. Biol..

[26] Park J-H (2007). RICK/RIP2 mediates innate immune responses induced through Nod1 and Nod2 but not TLRs. J. Immunol..

[27] Hugot JP (2001). Association of NOD2 leucine-rich repeat variants with susceptibility to Crohn’s disease. Nature.

[28] Bonen DK (2003). Crohn’s disease-associated NOD2 variants share a signaling defect in response to lipopolysaccharide and peptidoglycan. Gastroenterology.

[29] Ogura Y (2001). A frameshift mutation in NOD2 associated with susceptibility to Crohn’s disease. Nature.

[30] Inohara N (2003). Host Recognition of Bacterial Muramyl Dipeptide Mediated through NOD2: Implications for Crohn’s Disease. J. Biol. Chem..

[31] Girardin SE (2005). Identification of the Critical Residues Involved in Peptidoglycan Detection by Nod1. J. Biol. Chem..

[32] ter Horst R (2016). Host and Environmental Factors Influencing Individual Human Cytokine Responses. Cell.

[33] Oosting M (2016). Functional and Genomic Architecture of Borrelia burgdorferi-Induced Cytokine Responses in Humans. Cell Host Microbe.

[34] Li Y (2016). A Functional Genomics Approach to Understand Variation in Cytokine Production in Humans. Cell.

[35] Shah TS (2012). optiCall: a robust genotype-calling algorithm for rare, low-frequency and common variants. Bioinformatics.

[36] Gollob KJ, Viana AG, Dutra WO (2014). Immunoregulation in human American leishmaniasis: balancing pathology and protection. Parasite Immunol..

[37] Girardin SE (2003). Peptidoglycan Molecular Requirements Allowing Detection by Nod1 and Nod2. J. Biol. Chem..

[38] Girardin SE (2003). Nod2 Is a General Sensor of Peptidoglycan through Muramyl Dipeptide (MDP) Detection. J. Biol. Chem..

[39] Oosting M (2010). Recognition of *Borrelia burgdorferi* by NOD2 Is Central for the Induction of an Inflammatory Reaction. J. Infect. Dis..

[40] van Heel DA (2005). Muramyl dipeptide and toll-like receptor sensitivity in NOD2-associated Crohn’s disease. Lancet.

[41] Herskovits AA, Auerbuch V, Portnoy DA (2007). Bacterial Ligands Generated in a Phagosome Are Targets of the Cytosolic Innate Immune System. PLoS Pathog..

[42] Antonelli LRV (2005). Activated inflammatory T cells correlate with lesion size in human cutaneous leishmaniasis. Immunol. Lett..

[43] Oliveira F (2011). Lesion size correlates with Leishmania antigen-stimulated TNF-levels in human cutaneous leishmaniasis. Am. J. Trop. Med. Hyg..

[44] Khouri R (2009). IFN-beta impairs superoxide-dependent parasite killing in human macrophages: evidence for a deleterious role of SOD1 in cutaneous leishmaniasis. J. Immunol..

[45] Kobayashi KS (2005). Nod2-Dependent Regulation of Innate and Adaptive Immunity in the Intestinal Tract. Science..

[46] Homer CR, Richmond AL, Rebert NA, Achkar J, McDonald C (2010). ATG16L1 and NOD2 Interact in an Autophagy-Dependent Antibacterial Pathway Implicated in Crohn’s Disease Pathogenesis. Gastroenterology.

[47] Cooney R (2010). NOD2 stimulation induces autophagy in dendritic cells influencing bacterial handling and antigen presentation. Nat. Med..

[48] Dorta ML (2012). Improvements in obtaining New World Leishmania sp from mucosal lesions: Notes on isolating and stocking parasites. Exp. Parasitol..

[49] Laayouni H (2014). Convergent evolution in European and Rroma populations reveals pressure exerted by plague on Toll-like receptors. Proc. Natl. Acad. Sci..

[50] Battisti, J. M. & Minnick, M. F. Laboratory maintenance o*f Bartonella quintan*a. *Curr. Protoc. Microbiol*. Chapter 3, Unit 3C.1.1-3C.1.13 (2008). 10.1002/9780471729259.mc03c01s10 18729057

[51] Chomzynski P, Sacchi N (1987). Single-Step Method of RNA Isolation by Acid Guanidinium Thiocyanate–Phenol–Chloroform Extraction. Anal. Biochem..

[52] Morato CI (2014). Essential role of leukotriene B4 on Leishmania (Viannia) braziliensis killing by human macrophages. Microbes Infect..

