# Supplementary material for: The NOD2 receptor is crucial for immune responses towards New World Leishmania species
Source: Sci Rep. 2017 Nov 9;7:15219. doi: 10.1038/s41598-017-15412-7 (PMC5680260; doi:10.1038/s41598-017-15412-7)
Supplement: Supplementary file 1 — Supplementary information [file 41598_2017_15412_MOESM1_ESM.pdf]

The NOD2 receptor is crucial for immune responses towards New World *Leishmania* species

Jéssica Cristina dos Santos[1,2], Michelle S.M.A. Damen[1], Marije Oosting[1], Dirk J. de Jong[3], Bas Heinhuis[1], Rodrigo Saar Gomes[2], Carla Santos Araújo[4], Mihai G. Netea[1,5], Fátima Ribeiro-Dias[2*], Leo A.B. Joosten[1,2*]

Supplementary Table 1. Single-Nucleotide Polymorphisms (SNPs) evaluated in this study.

Gene	rs number	Mutation	Nucleotide change #	Amino acid change #
*NOD1**	-		Ins/Del ND1+32656	Glu796Lys
NOD2	rs2066847	Frameshift		Leu1007insC
NOD2	rs2066845	Missense	C>G	Gly908Arg
NOD2	rs2066844	Missense	C>T	Arg702Trp
NOD2	rs9302752	Unknown	C>T	Unknown
NOD2	rs7194886	Unknown	C>T	Unknown
NOD2	rs8057341	Unknown	A>G	Unknown
NOD2	rs2066847	Frameshift		3020insC

The first nucleotide (and corresponding amino acid) is the ancestral nucleotide and therefore is considered the wild-type allele.

*NOD1 insertion/deletion (Ins/Del) polymorphism ND1+32656, partially identified as rs6958571.

Supplementary Table 2. Primers sequence

GAPDH FW primer: 5’-AGG-GGA-GAT-TCA-GTG-TGG-TG-3’

GAPDH RV primer: 5’-CGA-CCA-CTT-TGT-CAA-GCT-CA-3’

NOD2 FW primer: 5’-CCC-TGC-AGC-TGG-ACT-ACA-ACT-3’

NOD2 RV primer: 5’-AGA-TGC-CTC-GGT-CTG-AGA-TAT-TG-3’

NOD1 FW primer: 5’-AGA-GGC-TCT-GCG-GAA-CCA-3’

NOD1 RV primer: 5’-TGT-GGA-GAT-GCC-GTT-GGA-3’

MATERIALS AND METHODS

Human embryonic kidney cell line (HEK)

HEK-293 cells were cultured in DMEM (Sigma) supplemented with 7.5% fetal bovine serum (Hyclone, Thermo Scientific), 100 U/mL penicillin and 100 μg/mL streptomycin (Invitrogen), at 37°C and 5% CO_2. Plasmid selecting agent for *NOD2*, 30 μg/mL blasticidin (Invivogen) and 100 μg/mL zeocine were added to the culture medium to ensure presence of this specific *NOD2* receptor in HEK-293 cells. Correct *NOD2* expression was confirmed by Reverse Transcription-polymerase chain reaction (RT-PCR). When 80% of cell confluence was reached, the HEK-293 cells were passaged, counted and used in stimulation experiments.

Evaluation of mRNA expression by quantitative real-time PCR (qPCR)

RNA was precipitated with isopropanol and washed with 75% ethanol followed by reconstitution in RNAse-free water. Subsequently, RNA was reverse transcribed into cDNA using the iScript cDNA synthese kit (Bio-Rad). Diluted cDNA was used for qPCR analysis that was performed with the use of the AB StepOnePlus polymerase chain reaction system (Applied Biosystems) with SYBR Green Mastermix (Applied Biosystems).

Evaluation of Macrophage infection

Under a light microscope (1000x), 300 cells were analysed and the percentage of infected cells and the mean number of intracellular parasites per infected cell were determined. Infection index = percentage of infected cells × mean number of parasites per infected cell.

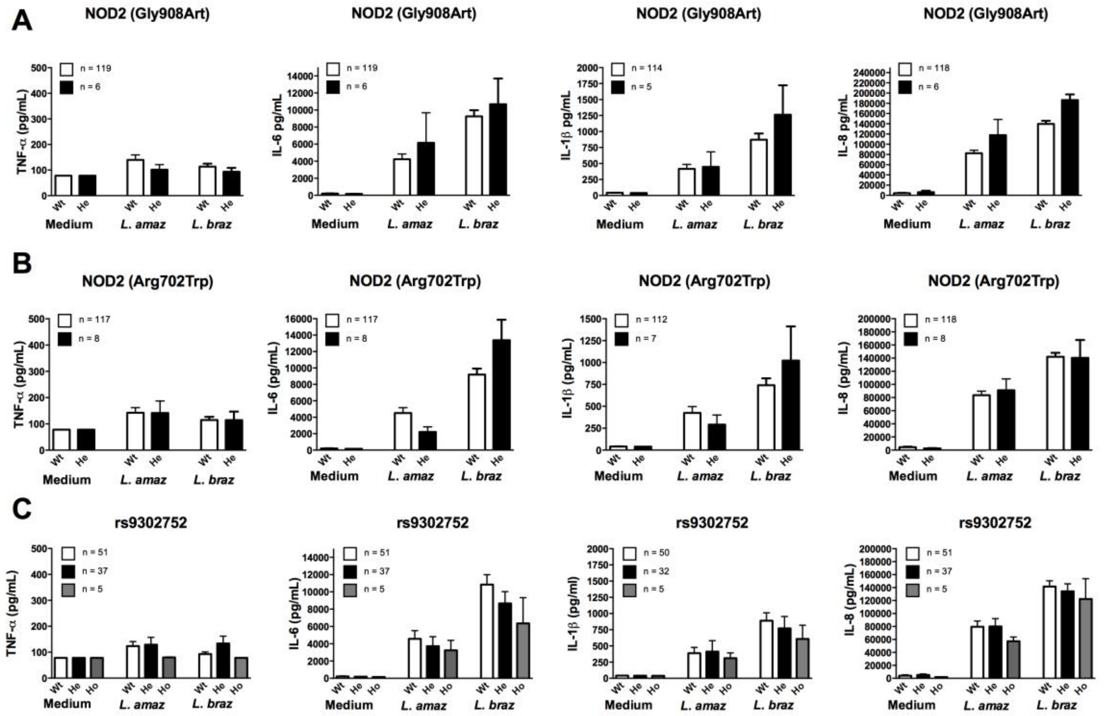

Figure supplementary 1: Peripheral blood mononuclear cells (PBMCs, 5 x 10^5 cells/100 μL) from healthy individuals genotyped for the *NOD2* were stimulated with different stimuli, including lysates of *Leishmania* parasites (50 μg/mL; *L.* (*L.*) *amazonensis*: *L. amaz*; *L.* (*V.*) *braziliensis*: *L. braz*). TNFα, IL-6, IL-1β and IL-8 concentrations were determined in supernatants by ELISA after 24 h of incubation. The results were stratified for the *NOD2* genotype. Bars represent individuals carrying no SNP (wild type, Wt, white bars), heterozygous SNP carriers (He, black bars), or homozygous variation (Ho, grey bars). Data represent the mean ± SEM.

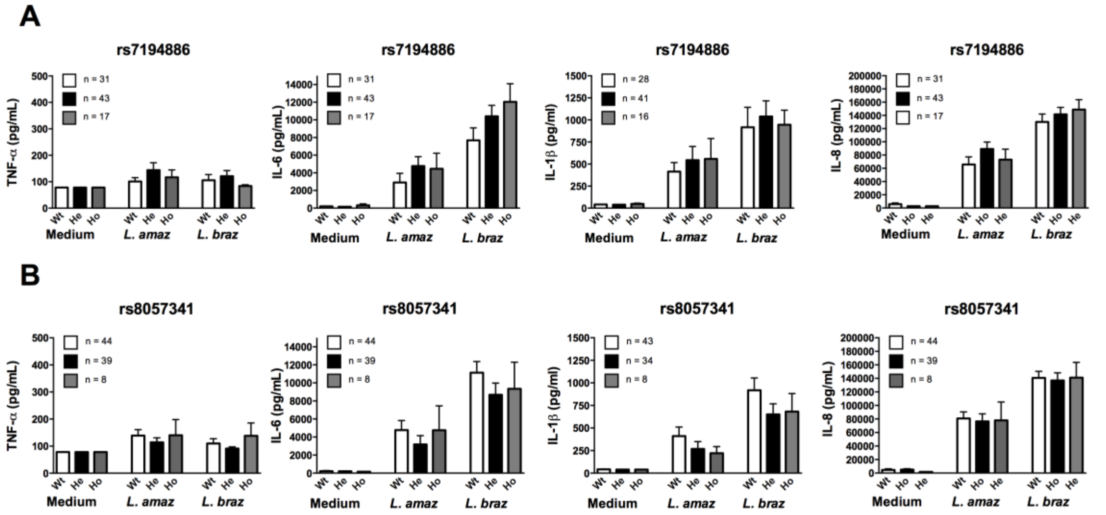

Figure supplementary 2: Peripheral blood mononuclear cells (PBMCs, 5 x 10^5 cells/100 µL) from healthy individuals genotyped for the *NOD2* were stimulated with different stimuli, including lysates of *Leishmania* parasites (50 µg/mL; *L.* (*L.*) *amazonensis*: *L. amaz*; *L.* (*V.*) *braziliensis*: *L. braz*). TNFα, IL-6, IL-1β and IL-8 concentrations were determined in supernatants by ELISA after 24 h of incubation. The results were stratified for the *NOD2* genotype. Bars represent individuals carrying no SNP (wild type, Wt, white bars), heterozygous SNP carriers (He, black bars), or homozygous variation (Ho, grey bars). Data represent the mean ± SEM.

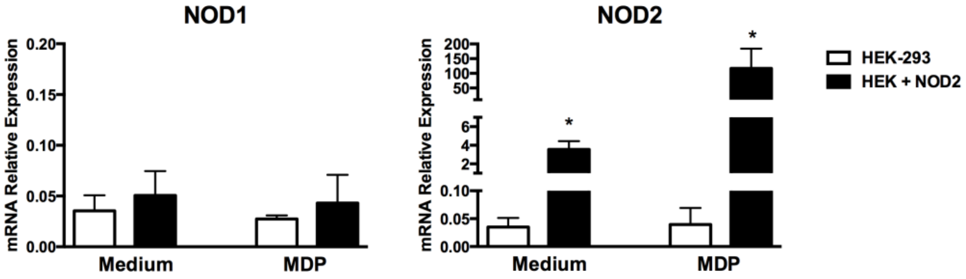

Figure supplementary 3: N*OD1* and *NOD2* mRNA expression in embryonic kidney (HEK)-293 cells (1 x 10^6 cells/100 µL) transfected or not with *NOD2*. Cells incubated in the absence (Medium) or presence of MDP (10 µg/mL) for 24 h. *NOD1* and *NOD2* mRNA expression were determined by quantitative real-time PCR. Data represent the mean ± SEM of three independent experiments, *p < 0.05; Unpaired t-test (HEK-293 vs HEK+NOD2).

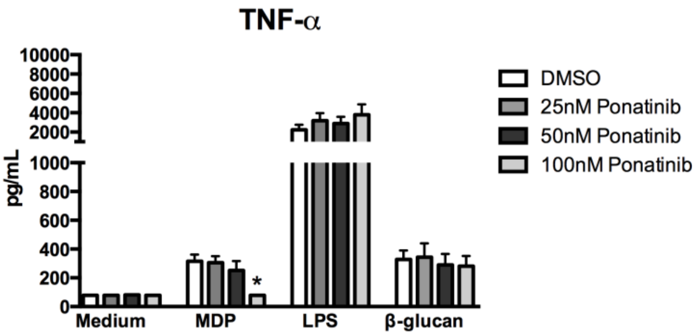

Figure supplementary 4: Peripheral blood mononuclear cells (PBMCs, 5 x 10^5 cells/100 µL) from healthy individuals were stimulated with MDP (10 µg/mL), LPS (10 ng/mL) or β−glucan (5 µg/mL) in the absence (white) or presence (grey) of the Ponatinib (25 nM; 50 nM and 100 nM); Medium (non-stimulated cells) and DMSO (vehicle) were included as negative controls. TNFα concentration was determined in supernatants by ELISA, after 24 h of incubation; Data represent the mean ± SEM, *p < 0.05; (Vehicle vs Ponatinib; n = 6, in 2 independent experiments done in duplicates, by Wilcoxon test).
